# RAD-seq reveals genetic structure of the F_2_-generation of natural willow hybrids (*Salix* L.) and a great potential for interspecific introgression

**DOI:** 10.1186/s12870-018-1552-6

**Published:** 2018-12-03

**Authors:** Susanne Gramlich, Natascha Dorothea Wagner, Elvira Hörandl

**Affiliations:** Department of Systematics, Biodiversity and Evolution of Plants (with Herbarium), University Goettingen, Untere Karspüle 2, 37073 Goettingen, Germany

**Keywords:** Population genomics, Hybrid evolution, Population structure, Sex chromosomes, Climate change

## Abstract

**Background:**

Hybridization of species with porous genomes can eventually lead to introgression via repeated backcrossing. The potential for introgression between species is reflected by the extent of segregation distortion in later generation hybrids. Here we studied a population of hybrids between *Salix purpurea* and *S. helvetica* that has emerged within the last 30 years on a glacier forefield in the European Alps due to secondary contact of the parental species. We used 5758 biallelic SNPs produced by RAD sequencing with the aim to ascertain the predominance of backcrosses (F_1_ hybrid x parent) or F_2_ hybrids (F_1_ hybrid x F_1_ hybrid) among hybrid offspring. Further, the SNPs were used to study segregation distortion in the second hybrid generation.

**Results:**

The analyses in structure and NewHybrids revealed that the population consisted of parents and F_1_ hybrids, whereas hybrid offspring consisted mainly of backcrosses to either parental species, but also some F_2_ hybrids. Although there was a clear genetic differentiation between *S. purpurea* and *S. helvetica* (F_ST_ = 0.24), there was no significant segregation distortion in the backcrosses or the F_2_ hybrids. Plant height of the backcrosses resembled the respective parental species, whereas F_2_ hybrids were more similar to the subalpine *S. helvetica*.

**Conclusions:**

The co-occurrence of the parental species and the hybrids on the glacier forefield, the high frequency of backcrossing, and the low resistance to gene flow via backcrossing make a scenario of introgression in this young hybrid population highly likely, potentially leading to the transfer of adaptive traits. We further suggest that this willow hybrid population may serve as a model for the evolutionary processes initiated by recent global warming.

**Electronic supplementary material:**

The online version of this article (10.1186/s12870-018-1552-6) contains supplementary material, which is available to authorized users.

## Background

Natural hybridization due to secondary contact has been observed in many plant and animal species. Especially in North America and Northern and Central Europe, a major driving force for secondary contact is the ongoing recolonization after the retreat of glaciers [1]. This process is amplified by human-induced global warming that also causes rapid range shifts of species [2–5], especially in mountain regions, leading to secondary contact of previously allopatric species [6]. The absence of strong pre- or postzygotic reproductive barriers may then lead to hybridization.

Many studies have investigated the evolutionary relevance of hybridization. Although there are some documented cases of homoploid hybrid speciation [7–9], speciation seems to be a rather rare outcome compared to the plenty of reported incidents of hybridization [10]. Important requirements for homoploid hybrid speciation seem to be strong ecological or geographical barriers that restrict gene flow between hybrids and the parental species [7, 11–13]. Thus, hybrid speciation is closely connected to the availability of novel or extreme habitats [14, 15]. Chromosomal rearrangements can also rapidly establish crossing barriers between parents and hybrids [9]. Generally, interspecific hybridization seems more likely to result in introgression than in speciation [7, 14]. In extreme cases, introgression can lead to genetic swamping threatening species integrity and posing a severe problem especially in small populations or rare species [16]. On the other hand, adaptive introgression can lead to the transfer of favourable alleles [10, 13, 17–19]. Introgression of favourable traits can increase the species’ genetic and phenotypic diversity, and hence the potential to adapt to novel environments [9, 20]. The outcome of an incipient hybridization event is not easy to predict because it depends on many factors like the fitness of the hybrids and their offspring [21], the impact of endogenous and exogenous selection, interactions of certain genotypes with the environment [22], and habitat availability [23].

To assess the evolutionary impact of a hybridization event, it is crucial to know the extent to which a genome is susceptible to the introgression of heterospecific alleles. Segregation distortion, the deviation from expected Mendelian segregation ratios, can be used as a measure of the resistance of the hybridizing species’ genomes to introgression [24, 25]. Further, it can be assumed that distorted loci are linked to genes that affect the viability or fitness of hybrids or their gametes [25, 26]. Thus, segregation distortion is also connected to reproductive barriers and the suppression of interspecific gene flow [27–29]. Segregation distortion can also arise from low recombination rates on sex chromosomes or in sex-determining regions [30, 31]. In dioecious plants, female-biased sex ratios are connected to segregation distortion at distorter loci [32]. The search for loci or regions under segregation distortion has therefore been applied, even in nonmodel species, as a basis to draw conclusions about the potential underlying causes of reproductive barriers between species [33–37], or to identify loci responsible for environmental adaptation [29, 38].

In this study, we investigate hybridization in a zone of secondary contact between two willow species, *Salix purpurea* L. and *S. helvetica* Vill., which are situated on the forefield of the Rhône Glacier in central Switzerland. *Salix helvetica* is a shrub that occurs naturally in the subalpine to alpine zone. *Salix purpurea*, on the other hand, is a widespread lowland species that was recently able to colonize higher altitudes due to global warming and subsequent glacier retreat [39]. Secondary contact and hybridization of these species takes place on glacier forefields that have recently become ice free. These glacier forefields are covered with sparse vegetation and offer plenty of space and different niches for the settlement of pioneer species like willows [39–41]. Hybridization even across sections and between distantly related species is a common phenomenon in *Salix* [42, 43]. Although *S. purpurea* and *S. helvetica* belong to different sections of the genus [44], they form natural hybrid zones in the European Alps [39]. The composition of such hybrid zones would provide important clues for an assessment of the evolutionary consequences of these hybridization events. A predominance of backcrosses would render introgression of genes between the parental species more likely, whereas the domination of F_2_ hybrids (i.e. F_1_ hybrid x F_1_ hybrid) might be an indication of the potential for hybrid swarm formation and further hybrid evolution. In an earlier study on the willow population at the Rhône Glacier, an attempt has been made to determine the exact class of the hybrids (F_1_, F_2_, backcrosses) on the glacier forefield based on genotyping with microsatellite markers [39]. These markers clearly separated the two parental species and confirmed the hybrid origin of phenotypically intermediate individuals, but their resolution was not sufficient for an unequivocal assignment of all individuals to a certain hybrid class [39]. Thus, the precise composition of the hybrid zone is still uncertain. However, we found that the hybrids between *S. purpurea* and *S. helvetica* are fertile and produce viable seeds in the natural population, and thus confirmed that hybridization can proceed beyond the F_1_ hybrid generation [45]. The offspring raised from these naturally formed seeds offered the opportunity to study not only progeny classes of second generation hybrids, but also putative segregation distortion and phenotypic traits.

In order to overcome the limitations caused by a low number of markers, we used restriction-site associated DNA sequencing (RAD-seq) to generate a genome-wide set of thousands of single-nucleotide polymorphisms in this nonmodel species. High-quality biallelic SNPs were used to (i) determine the class of the hybrids on the glacier forefield and of offspring produced by F_1_ hybrids in order to predict the consequences of this hybridization event. Further, (ii) we were looking for deviations from expected segregation patterns at individual loci in F_2_ hybrids and backcrosses to determine if alleles of one parental species were favoured over the other. Population genomic analyses were accompanied by morphometric measurements to (iii) get insights into the variation of a selected, potentially adaptive phenotypic character (plant height) in the respective second generation hybrid classes.

## Results

### RAD-seq and SNP calling

RAD-seq of *S. purpurea*, *S. helvetica* and their hybrids yielded an average of 7.5 × 10^6^ reads per individual (SD 2.4 × 10^6^). The average per base sequence quality was very high with a Phred score of 40 for all positions in the reads in all samples. The average depth of read coverage was 59x (SD 18). The stacks-pipeline initially generated 49,081 loci. After the application of all filters, 5758 nuclear loci remained. After mapping the loci to the plastid genome no match was observed. However, of the filtered loci 933 reads aligned to coding regions in the genome of *P. trichocarpa*. The results of population genetic and progeny analyses did not change when the SNPs lying in coding regions were excluded, and thus we performed all analyses with all 5758 SNPs.

### Population genetic structure

**Fig. 1:**
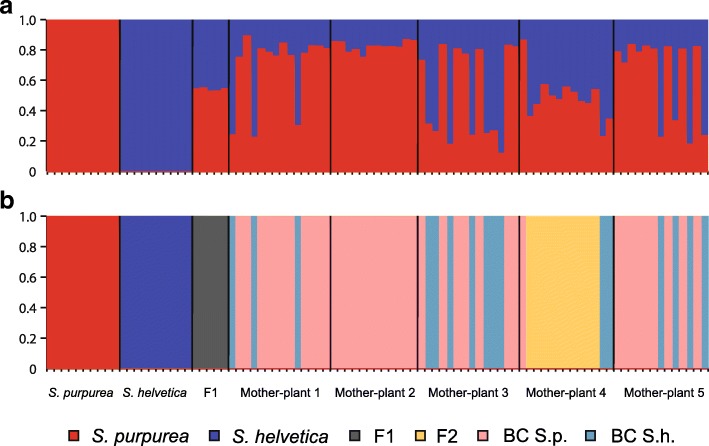
Genetic structure of parental species and their hybrids. Admixture proportions (q_i_) based on two clusters (*K* = 2) estimated by structure (**a**) and assignment of hybrid class in NewHybrids (**b**). The plots include the five F_1_ mother-plants representing all 45 F_1_ hybrid individuals in the sample (for admixture proportions and assignment of hybrid class of the remaining 40 hybrids see Additional file 1: Table S1 and S2). The offspring of the mother-plants is sorted by mother-plant. The order of the individuals within the different groups is the same in both plots

**Fig. 2:**
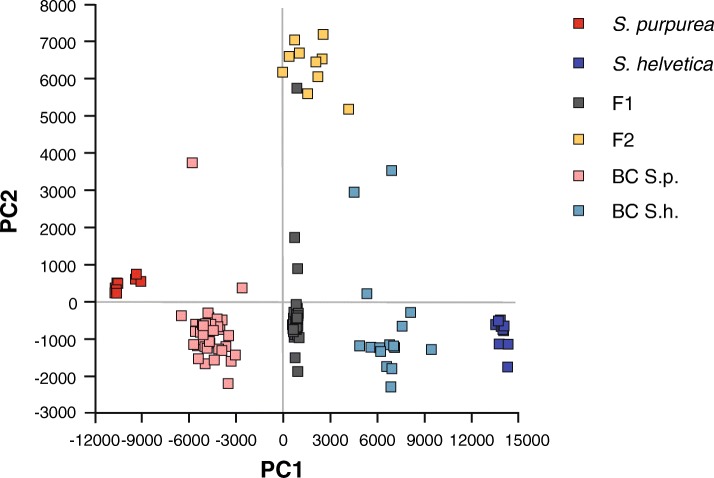
Principal coordinates analysis depicting the pairwise genetic distances among the 133 individuals in the sample. The first axis accounted for 12.7% of the total variance and the second axis accounted for 1.52%. BC, backcross; S.p., *S. purpurea*; S.h., *S. helvetica*; F_2_, F_1_ x F_1_ cross

The structure analysis confirmed that the most likely number of populations (*K*) in the sample was two (Additional file 1: Figure S1). It can thus be assumed that all hybrids were crosses between *S. purpurea* and *S. helvetica* with no third species involved (Fig. 1a). The F_ST_ value between *S. purpurea* and *S. helvetica* was 0.53 for the filtered loci, but only 0.24 when all 49,081 unfiltered loci were considered, indicating a strong dependence of F_ST_ values on locus selection. The NewHybrids analysis confirmed the parental classes and revealed that all hybrid individuals sampled on the glacier forefield of the Rhône Glacier were F_1_ hybrids. Although the dataset had to be restricted to 300 loci because NewHybrids cannot handle a larger number of loci, the results for the assignment to the different classes had 100% posterior probability support for all individuals. Accordingly, *S. purpurea* and *S. helvetica* were clearly separated along the first axis in the PCoA (Fig. 2) with the hybrid individuals sampled at the Rhône Glacier clustering exactly in the middle along the second axis.

The offspring of the F_1_ hybrids consisted of backcrosses to *S. purpurea*, backcrosses to *S. helvetica*, and of F_2_ hybrids (crosses between F_1_ hybrids). F_2_ hybrids were only observed among the offspring of one of the five mother-plants (Fig. 1b). All other plants produced backcrosses in both directions. Overall, significantly more backcrosses to *S. purpurea* (*n* = 40) than to *S. helvetica* (*n* = 16) were detected (binomial test, 2-sided, *p* = 0.001, *n* = 57). The different hybrid classes also formed well-separated clusters in the PCoA analysis (Fig. 2). The backcrosses clustered in the respective parental half without overlapping with the purebred individuals. The F_2_ hybrids formed a cluster of their own, clearly separated from the other individuals along the second axis. The F_1_ individual that clusters with the F_2_ hybrids is the mother of the F_2_ hybrids.

### Segregation distortion in the second generation hybrids

The filtering of all loci for F_ST_ = 1 between *S. purpurea* and *S. helvetica* retrieved 396 species-specific SNPs, which included SNPs in coding regions of the genome. Of these, 334 loci could be aligned to the *S. purpurea* genome. The alignment showed that they were represented on all 19 chromosomes of the *Salix* genome (Table 1).

**Table 1:** Distribution of 334 species specific SNPs detected in *S. purpurea* and *S. helvetica* on the 19 chromosomes of *S. purpurea*

Chromosome	Nr of loci
I	26
II	37
III	24
IV	9
V	26
VI	35
VII	16
VIII	21
IX	25
X	35
XI	6
XII	5
XIII	19
XIV	18
XV	2
XVI	20
XVII	3
XVIII	5
XIX	2

There were no significant deviations from the expected segregation patterns neither in the backcrosses nor in the F_2_ hybrids. Although the distribution of homozygous and heterozygous genotypes was skewed at some loci in the F_2_ hybrids (Additional file 2: Table S3), the number of individuals seemed to be too low to support significant deviations.

### Plant height in the second generation hybrids

**Fig. 3:**
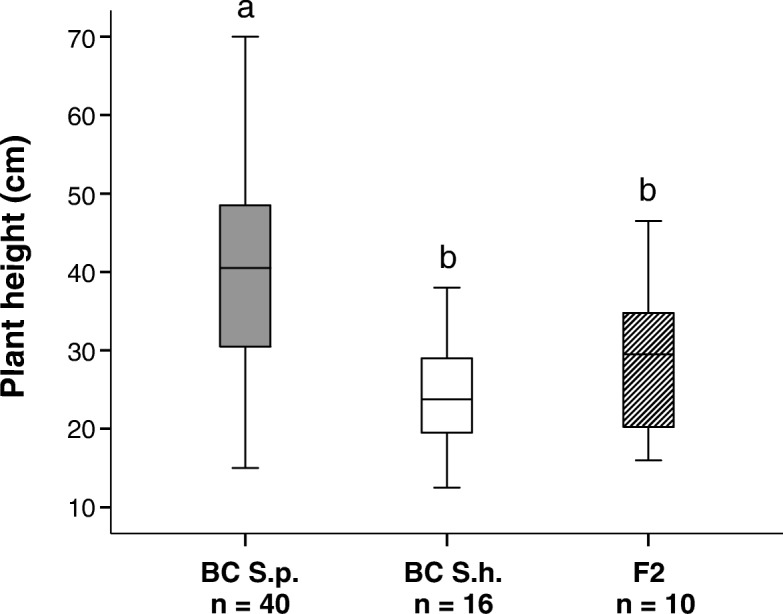
Boxplots of the plant height of backcrosses to *S. purpurea* (BC S.p.), backcrosses to *S. helvetica* (BC S.h.), and F_2_ hybrids (F_1_ x F_1_). Significant differences between groups are indicated by letters. The median is indicated by the black horizontal line. The bottom and top of the boxes show the 25 and 75 percentiles, respectively. The whiskers extend to the highest and lowest value that is not an outlier

The one-way ANOVA revealed significant differences in plant height between the three groups (F_2,67_ = 15.064, MS = 219.71, *p* < 0.001, Fig. 3). Backcrosses to *S. purpurea* ranged from 15 to 70 cm (M = 41.8 cm, SD = 14.2) and were significantly taller than backcrosses to *S. helvetica* (12.5–38 cm, M = 24 cm, SD = 6.3) or F_2_ hybrids (16–46.5 cm, M = 29.4 cm, SD = 10.2).

## Discussion

### Composition of the natural hybrid population

Our study confirms the existence of a natural secondary contact hybrid zone of *S. helvetica* and *S. purpurea* on the Rhône glacier forefield. RAD-seq data enabled a much better resolution of the hybrid classes than the microsatellite markers applied in our previous study [39]. While the results based on genotyping with microsatellites had suggested that two-thirds of the hybrids were probably later generation hybrids (F_2_ hybrids or backcrosses), the analysis of the same hybrids with RAD-seq data showed with maximum statistical support that all hybrid individuals sampled on the forefield of the Rhône Glacier were F_1_ hybrids. This discrepancy in the hybrid classification is probably due to the low number of microsatellite loci used in the previous study that had to be restricted to primers amplifying in both species. Further, the lack of species-specific alleles at the microsatellite loci made it difficult to determine from which species an allele was inherited in the admixed genotypes. Similar discrepancies in the classification of hybrids were also observed in studies on *Populus* hybrid zones. While no hybrids were classified as F_1_ hybrids based on genotyping with microsatellites [46], subsequent genotyping-by-sequencing revealed that most hybrids belonged to the F_1_ generation [47]. The authors explained the different results with the shortcomings of microsatellites, like allele dropout [47]. We think that the results based on RAD-seq data are more reliable due to the large number of loci that produce a much higher resolution than the DNA fingerprinting techniques used so far [48]. Further, all hybrids were assigned to the respective hybrid class with 100% posterior probability in the RAD-seq analysis, whereas the SSR analysis gained less than 95% posterior probability for many individuals. Altogether RAD-seq clearly performs better for studies on interspecific hybridization than microsatellites.

We expected to find some later generation hybrids on the glacier forefield because the analysis of offspring grown from seeds that had been collected from naturally pollinated F_1_ hybrids at the forefield of the Rhône Glacier [45] suggested that backcrosses and F_2_ offspring can be regularly formed in this population. However, the results revealed only F_1_ generation hybrids. We assume that this lack of second generation hybrids is due to the sampling strategy. We included only material from adult individuals because the leaves, flowers and fruits of juvenile plants and seedlings did not yet show the typical phenotypic characteristics of adult willows. In a previous study, we already concluded that the hybrid population on this recently emerged glacier forefield is probably not more than 20–30 years old [39]. We thus believe that second generation hybrids on the glacier forefield were not yet present or very rare in the adult generation during our sampling. In order to clarify whether backcrosses and F_2_ hybrids have meanwhile grown up in the natural population, an extended sampling strategy that includes juvenile plants and a broad representation of phenotypes would have to be applied.

We have no reason to assume that backcrosses to either parent as well as F_2_ hybrids cannot establish on the glacier forefield. In a study on hybrid fertility, we found that the seed output of hybrids was reduced compared to the parents, but that seeds showed high germination ability, and seedlings developed well [45]. Thus it seems unlikely that there are no backcrosses or F_2_ hybrids on the glacier forefield, although their numbers may probably be still lower compared to F_1_ hybrids. Alternatively, habitat mediated selection may act against the establishment of later generation hybrids on the parental sites, as it has been observed in *Rhododendron* hybrid zones [49]. At the time the first F_1_ hybrids were formed, the glacier forefield was still in an early state of succession with less vegetation cover so that the conditions were more favourable for the establishment of willows, which are pioneer species. Later in time, when the backcrosses and F_2_ hybrids were produced, the vegetation may have been denser so that the conditions for the establishment may have become more difficult. However, it should also be kept in mind that glacier retreat is ongoing and that open pioneer sites for colonization will be continuously available. Although we did not find later generation hybrids in our present sampling, it may still be concluded that the hybrid population on the glacier forefield is able to develop beyond the F_1_ generation so that hybridization may have further consequences as discussed in the next section.

### Classes of hybrid offspring

In contrast to the findings made in the natural population on the glacier forefield, the offspring raised from seeds formed in the wild consisted of F_2_ hybrids (F_1_ crossed with F_1_) and backcrosses to both parental species. Interestingly, F_2_ hybrids were only found among the offspring of one of the five mother-plants. On the glacier forefield, this female plant stands less than three metres away from a male F_1_ hybrid (S. Gramlich, unpublished observation). It thus seems that F_2_ hybrids are only produced in high numbers, when male and female F_1_ hybrids stand close together. This arrangement is quite rare on the glacier forefield so that female F_1_ hybrids are closer to male individuals of *S. purpurea* or *S. helvetica* in most cases (S. Gramlich, unpublished observation). The parental species and the hybrids occur evenly dispersed over the whole area so that there is no spatial structure like a clumped distribution or a cline from one parental species to the other [39]. Therefore it is more likely that female F_1_ hybrids are pollinated by one of the purebred species so that they will produce backcrosses. Accordingly, our results showed that the offspring of the sampled F_1_ hybrids consisted mainly of backcrosses to the parental species and only few F_2_ hybrids. Similar findings were made in poplars where purebred female plants produced exceptionally large amounts of backcross seedlings when they were surrounded by F_1_ hybrids [50]. Overall, there were more backcrosses to *S. purpurea* than to *S. helvetica* in the sample, yet the reason for this result is unclear. Possible causes could be a greater overlap of flowering time between *S. purpurea* and the hybrids, postzygotic selection against backcrosses to *S. helvetica*, stochastic factors like a closer proximity between male *S. purpurea* and female F_1_ hybrids than between *S. helvetica* males and F_1_ hybrids, or sampling bias due to the choice of mother plants in the analysis, the limited number of progenies, or conditions for pollination in the year of sampling. Another possible interpretation could be that pollen limitation is stronger for pollen of *S. helvetica* than for pollen of *S. purpurea*. Accordingly, a reduced seed set was found in purebred *S. helvetica* compared to purebred *S. purpurea* [45]. The seed set in willows is pollen limited [51], and thus the pollen of *S. helvetica* could also be transported less efficiently. Pollen-pistil incongruences also represent a strong prezygotic crossing barrier in willows [52]. Pollen tube growth could act differentially between *S. purpurea* and *S. helvetica*, as the former species has a much shorter style and a capsule without a beak, while the latter has long styles and beaked capsules. However, the determination of the exact causes of the observed pattern requires further research. Irrespective of the direction of backcrossing it can be concluded that, at least in this early stage of secondary contact, a higher production of backcrosses than of F_2_ hybrids renders a future trajectory of introgression more likely than hybrid speciation.

### Second generation hybrids show no signs of segregation distortion

This is one of the first studies on nonmodel plant species where segregation distortion was analyzed with RAD-seq data. The power of this marker system for detecting segregation distortion and linkage groups was demonstrated e.g. on hybrid fish [29] and on white cypress pine [38]. The fact that there are only two alleles per locus makes it difficult to determine the species of origin of an allele in a hybrid individual, especially when the alleles are evenly distributed in the parental species. Therefore we restricted our analyses to species-specific loci. We believe that this subsampling is representative of genome-wide patterns of hybridization because the markers are located on all 19 chromosomes of the *Salix* genome.

We did not detect significant deviations from the expected Mendelian segregation ratios in the F_2_ hybrids or backcrosses. It is expected that the magnitude of distorted loci correlates with the level of divergence between the parental species [53]. The divergence between *S. purpurea* and *S. helvetica* turned out to be quite low with a F_ST_ value of 0.3 in our study based on microsatellite makers [39]. Divergence based on RAD-seq loci is also quite low with an F_ST_ value of 0.24 for completely unfiltered loci, but moderate for the filtered loci (F_ST_ = 0.53). This increase of F_ST_ is thought to be due to the removal of loci that do not discriminate the parental species. Thus we think that the low F_ST_ value based on the unfiltered loci gives a more realistic estimate of the population divergence. Another hint for the low divergence between *S. purpurea* and *S. helvetica* is that they hybridize easily although they belong to different, unrelated sections or clades of the genus *Salix* [44, 54]. However, phylogenetic studies showed in general a low genetic divergence between species and sections in the genus *Salix*, especially in the shrub species [55–57]. Recently, the phylogeny of the whole subgenus comprising the shrub willows could be resolved using RAD sequencing while more conservative markers had failed [54]. Thus, a low genetic divergence between the parental species seems to be a likely explanation for the absence of segregation distortion in the hybrids.

Genetic incompatibilities that cause hybrid sterility or inviability and thus act as postzygotic reproductive barriers accumulate with evolutionary divergence of the parental species [58, 59]. We observed that F_1_ hybrids produced less seeds than *S. purpurea* or *S. helvetica* but that the seeds they produced were viable and developed equally well as seedlings from the purebred species [45]. Due to the shallow genetic divergence of the parental species there seems to be a certain degree of postzygotic (i.e. intrinsic) selection before seed maturation during meiosis, pollination, fertilization or seed development, but the absence of segregation distortion suggests that heterospecific alleles are not selectively purged. Because large parts of the genome seem to be unaffected by segregation distortion, it can be assumed that the genome is susceptible to introgression, as it was also concluded for backcrosses in *Iris* [25].

Another interesting finding is that only two species-specific loci are located on chromosome XV that carries the sex determination locus in *Salix* [60]. Other studies found that sex chromosomes were highly divergent due to suppressed recombination and the accumulation of species-specific differences [30]. However, in *Salix*, as well as in *Populus*, no heteromorphic sex chromosomes have been discovered yet. Stölting et al. [61] did also not identify fixed SNPs between *Populus* species on chromosome XIX that carries the sex determining locus in *Populus* [62–64]. They concluded that, against the predictions, the incipient sex chromosome of *Populus* is not resistant to gene flow and introgression. Accordingly, Macaya-Sanz et al. [65] also detected gene flow on chromosome XIX in *Populus*. This finding seems to be reflected in *Salix* due to the low number of species-specific SNPs on chromosome XV detected in this study.

In contrast to the genomic data, segregation became obvious in phenotypic traits in one-year old juvenile plants. *Salix helvetica* is a shrub that reaches a height of ca. 50–80 cm [43]. *Salix purpurea* can reach up to 6 m in the lowland [66] but on the glacier forefield the shrubs were ca. 160–180 cm high (S. Gramlich, unpublished observation). With respect to plant height, the backcrosses seem to keep the traits of the recurrent parent, as expected, whereas the F_2_ hybrids adopted the lower height of *S. helvetica,* even in the absence of external selection under equal garden conditions. This is striking because a typical feature for alpine shrubs is the reduction of plant height (typically < 50 cm) as the plants are better protected by snow cover during freezing periods [54, 67]. Growth height thus appears to be a promising candidate for studying an adaptive trait in this hybrid system.

## Conclusions

Range shifts initiated by climate change will increase the likelihood of secondary contact hybridization in some species [68]. Comparisons of the outcome of diverse hybridization events induced by climate change are important in order to assess the effects of such events on biodiversity so that conservation measures can be initiated if necessary [69]. Which effect will the hybridization event have on the genetic diversity of the hybridizing willow species? We found that introgression is highly likely because intrinsic barriers against hybridization and gene flow between *S. purpurea* and *S. helvetica* are low. Further, hybrids and the purebred species occur in a mixed stand on the glacier forefield leading to a continuing formation of F_1_ hybrids and backcrosses. Introgression might be asymmetric because there were more backcrosses to *S. purpurea* in the sampling. Due to the isolated location, introgression might be highly localized affecting mainly the gene pools of *S. purpurea* and *S. helvetica* individuals on the glacier forefield and the surrounding slopes. On the other hand, we already discovered another population of hybrids between *S. purpurea* and *S. helvetica* at a higher altitude on the Morteratsch glacier [39], and thus it can be assumed that further hybrid populations will emerge at other locations in the European Alps due to the ongoing retreat of glaciers. Many localized, independent hybridization events would make a wider distribution of introgressed alleles more likely.

In general, hybridization appears to increase genetic and phenotypic variability in the offspring population. Interspecific exchange of genes via introgression is considered to be an important evolutionary force because it may lead to the transfer of adaptations [70]. Adaptation is viewed as the most important process that promotes divergence during speciation [13, 71]. In this way, introgression of adaptive traits could lead to the formation of ecotypes or even new species [70]. However, the long generation turnover of shrubs and the time needed to establish populations makes it difficult to predict the adaptive value of traits. Long term monitoring of such hybrid populations is essential to draw final conclusions.

Willow hybrids may also serve as models for the evolutionary processes initiated by global warming. Due to their properties as pioneer species, range shift and establishment of willows in novel habitats may be more rapid than in other species. The observations made in this model system may thus help to anticipate evolutionary processes that might affect species with lower dispersal rates much later in time.

## Methods

### Sampling

Leaf samples were collected at the forefield of the Rhône Glacier in central Switzerland (46°34′03.0″N, 08°22′12.3″E) from a mixed stand of *S. purpurea, S. helvetica*, and their hybrids. All plant samples were collected with the permission of the Canton du Valais, Service des forêts et du paysage. All individuals have already been genotyped at nine microsatellite loci in a previous study, which also included some reference populations sampled outside the glacier forefield [39]. For the present study, we sampled leaves from six individuals of *Salix purpurea* and nine individuals of *S. helvetica* from the glacier forefield. To extend the data of the purebred species, we also included four individuals of *S. purpurea* from three additional locations in Germany (51°18′53.0″N, 11°54′19.8″E, 51°44′32.2″N, 10°43′31.8″E; 49°21′13.0″N, 8°14′15.0″E) and one *S. helvetica* individual from Austria (46°49′21.6″N, 10°59′25.0″E).

We included a comprehensive sampling of 45 hybrids from the glacier forefield. These plants were classified as hybrids by both an intermediate phenotype between the parental species and genetic analysis that had been conducted in a previous study [39]. The identification of all specimens was done by S. Gramlich. Herbarium vouchers of each purebred and hybrid sample were deposited in the herbarium of the University of Göttingen (GOET).

Among these 45 hybrids, we selected five hybrids that had a > 95% probability of being a F_1_ hybrid in the NewHybrids analysis of our previous study [39]. To investigate the second hybrid generation, seeds that had been collected from these five naturally pollinated F_1_ hybrids at the forefield of the Rhône Glacier were germinated under controlled conditions (for details see [45]). Seedlings were grown for 1 year under equal conditions in climate growth chambers (see below). Out of hundreds of juvenile plants, a subset of 20–30 progenies per mother plant was selected that represented the phenotypic diversity among the offspring. From each of five F_1_ mother plants 13–14 progeny (overall *n* = 68) were finally sampled for RAD-seq analysis. The five mother plants from the natural population at the Rhône Glacier were also included in the sampling. The final dataset for RAD-seq analysis comprised 133 individuals.

### DNA extraction, RAD-seq

DNA was extracted from silica-dried leaves using the DNeasy Plant Mini Kit (Qiagen, Hilden, Germany) following the manufacturer’s protocol. The DNA concentration was assessed with a Qubit 3.0 fluorometer (Thermo Fisher Scientific, Waltham, MA, USA) and the samples were normalized to a concentration of 30 ng/μl. Aliquots of 3 μg DNA were then submitted to Floragenex Inc. (Eugene, OR, USA) for library preparation and single-end RAD sequencing (following the protocol of [72]). The total genomic DNA was digested with the restriction enzyme *Pst*I. The size selection of 300 bp – 500 bp with a Pippin Prep (Sage Science, Beverly, MA, USA) was followed by the ligation of sequencing adaptors and unique 10 bp barcodes for each sample. The samples were sequenced on an Illumina HiSeq 2500 Instrument (Illumina Inc., San Diego, CA, USA) and raw reads were delivered in FASTQ format trimmed to 100 bp.

### Bioinformatic analysis of raw data and SNP calling

The software stacks v. 1.44 [73, 74] was used for demultiplexing, SNP discovery, and genotyping. First, we demultiplexed the raw reads and removed low-quality reads using the *process_radtags* program implemented in stacks with the default parameters. In this step, the 10 bp barcodes were removed from the reads so that the final length of the reads was shortened to 90 bp. Afterwards, the quality of each sample was assessed with FastQC v. 0.11.4 [75]. Loci were assembled de novo using the *denovo_map* pipeline that merges RAD-tags into loci in each sample (*ustacks*), creates a catalog containing the merged loci from multiple samples (*cstacks*), and finally matches the loci from each sample against the catalog (*sstacks*). The first step within the pipeline is the creation of so called stacks (matching reads) out of the raw reads of each individual. The stacks provide the basis for building loci [73]. The minimum number of matching raw reads (minimum depth of coverage) required to create a stack (*m*) was set to 10. Thus, calling of heterozygotes requires at least 10 reads of each allele. The maximum number of nucleotide mismatches allowed between two stacks was set to 5 both for processing loci within individuals (*M*) and between individuals (*n*) when building the catalog. Appropriate values for *M* and *n* were determined in preliminary test runs. In these runs, *M* and *n* had the same value set between 2 and 7, and the total number of loci as well as the number of polymorphic loci was recorded. Following the recommendations of Viricel et al. [76] we chose the set of parameters where the total number of loci as well as the number of polymorphic loci reached an asymptote. Finally we used the *populations* program in stacks to extract loci that were present in all three groups (*S. purpurea*, *S. helvetica*, hybrids) in at least 70% of the individuals in each group (*r*). Data analysis was restricted to the first SNP at each locus in order to obtain unlinked SNPs required for population genetic analysis. Plink v 1.0.7 [77] was used to filter out SNPs with a minor allele frequency < 0.05, and SNPs that were out of the Hardy–Weinberg equilibrium at *p* < 0.05 in one or both parental populations. Further, individuals with a genotyping rate < 90% were also excluded from the analysis leading to the exclusion of two individuals of the hybrid offspring. We also applied further filters to remove potentially paralogous loci resulting from the recent ‘salicoid’ duplication event [78]. Collapsed paralogous copies at such loci should be characterized by an excess of heterozygosity and an increased coverage depth [79]. Thus, we removed loci with an observed heterozygosity (H_o_) ≥ 0.6, F_IS_ < 0, or a coverage depth that was greater than twice the standard deviation. Further, we also removed loci with a F_ST_-value of 0 between *S. purpurea* and *S. helvetica* in order to restrict the analyses to loci with variation between the parental species. H_o_, F_IS_, and F_ST_-values for each locus were generated by the *populations* program. The filtered loci were aligned to the *Populus trichocarpa* genome [78] to detect SNPs that were in coding, putative highly conservative regions, and to the plastome of *Salix suchowensis* [80] (GenBank: KM983390.1) to remove maternally inherited plastid markers. However, since no match to the plastid genome was observed, all loci appear to belong to the nuclear genome. Alignments were performed in Geneious R 10.0.9 [81]. After the filtering, 5758 loci remained for population genomic analysis.

### Genetic structure of the hybrid zone and the progenies

Pairwise genetic distances between individuals were calculated using the genetic distance measure of Smouse and Peakall [82] implemented in the R package *PopGenReport* [83]. Based on these genetic distances we performed a PCoA implemented in the R package *ade4* [84]. We used the program structure v. 2.3.4 [85] to confirm that all hybrids originated from crosses between *S. purpurea* and *S. helvetica* without the involvement of a third species. All 5758 loci were included in the structure analysis. We tested *K*-values ranging from 1 to 7 without prior population information under the admixture model assuming independent allele frequencies. Five runs were performed per tested *K*-value and the most likely *K*-value was determined using the method of Evanno et al. [86]. Finally, we determined the parental plants and the hybrid categories (F_1_, F_2_, backcrosses) of each hybrid individual with the program NewHybrids [87]. We designated only the hybrid x hybrid class as F_2_ hybrids, while the progeny as a whole was called second generation hybrids. Due to the young age of the hybrid zone on the forefield (approximately 20–30 years) it can be assumed that all individuals still belong to the early hybrid generations so that an assignment to exact hybrid classes is possible. NewHybrids cannot handle large datasets and thus we restricted the dataset to the first 300 loci of the whole dataset. We also ran NewHybrids with 300 loci that were selected randomly to ensure that the patterns were consistent across different subsets of the genome. The results were the same but we chose to use the first 300 loci to ensure the reproducibility of the results. Structure and NewHybrids were run using a burn-in period of 10,000 followed by 50,000 MCMC iterations. Longer run times were tested using reduced data sets, but did not change the results substantially.

### Analysis of segregation distortion in the second generation hybrids

We selected loci showing fixed differences between *S. purpurea* and *S. helvetica* for the analysis of segregation distortion so that the origin of an allele in a hybrid individual could be unequivocally determined. We checked whether all 45 F_1_ hybrids were heterozygous at these loci. Overall, 396 loci met both criteria. The R package *introgress* [88] was used to count the number of alleles derived from each of the parental populations for each hybrid individual at each locus. These counts were used as the basis to detect deviations from the expected segregation patterns. In the F_2_ hybrids, we tested for the deviation from the expected 1:2:1 distribution of homozygous and heterozygous genotypes found at each locus. In the backcrosses, we tested for the deviation from the expected 1:1 distribution of homozygous and heterozygous genotypes. χ^2^ goodness-of-fit tests were performed in R [89]. In the analysis of the F_2_ hybrids, *p*-values were computed by Monte Carlo simulation due to the low number of individuals. We corrected for multiple testing using the false discovery rate (FDR) method of [90] with α = 0.10.

We performed a Blast search of all RAD-loci containing species specific SNPs against the *S. purpurea* genome using Phytozome 12 [91] to determine on which chromosomes the loci were located. The alignment was accepted when the reads showed > 98% identity over the whole read length of 90 bp.

### Evaluation of plant height

The seedlings grown from five naturally pollinated F_1_ hybrids at the forefield of the Rhône Glacier [45] were raised in climate chambers for about 1 year at 18 °C with a 16-h light period (c. 250 μmol m^− 2^ s^− 1^) under equal soil and watering conditions. At this age, plants had leaves typical for adults (for some examples see Additional file 1: Figure S2), but did not yet produce flowers. Before these juvenile plants were transferred to pots for outdoor cultivation, the length of the longest shoot was measured to the nearest 0.5 cm. A one-way ANOVA was performed to test for differences between groups (according to the NewHybrids analysis: F_2_, backcross to *S. purpurea*, backcross to *S. helvetica*), followed by the Games–Howell test as post hoc test. The type I error rate was α = 0.05. The ANOVA was performed using SPSS version 24 (IBM Corp., Armonk, NY).

## Additional files

Additional file 1: Supporting information for the Methods and Results section. Contains **Figure S1.** plot of the ∆*K*-values for the range of *K*-values tested in structure, **Figure S2.** examples of the leaf shape of *S. purpurea*, *S. helvetica* and their hybrids, **Table S1.** admixture proportions of 40 F_1_ hybrids, **Table S2.** assignment of hybrid class of 40 F_1_ hybrids. (PDF 288 kb)

Additional file 2: **Table S3.** Distribution of homozygous and heterozygous genotypes found at each locus in the F_2_ hybrids and distribution of parental alleles in the backcrosses. (XLSX 29 kb)
