# Supplementary material for: RAD-seq reveals genetic structure of the F2-generation of natural willow hybrids (Salix L.) and a great potential for interspecific introgression
Source: BMC Plant Biol. 2018 Dec 3;18:317. doi: 10.1186/s12870-018-1552-6 (PMC6276181; doi:10.1186/s12870-018-1552-6)
Supplement: Supplementary file 1 — Supporting information for the Methods and Results section. Contains Figure S1. plot of the ∆K-values for the range of K-values tested in structure, Figure S2. examples of the leaf shape of S. purpurea, S. helvetica and their hybrids, Table S1. admixture proportions of 40 F1 hybrids, Table S2. assignment of hybrid class of 40 F1 hybrids. (PDF 288 kb) [file 12870_2018_1552_MOESM1_ESM.pdf]

Supporting Information

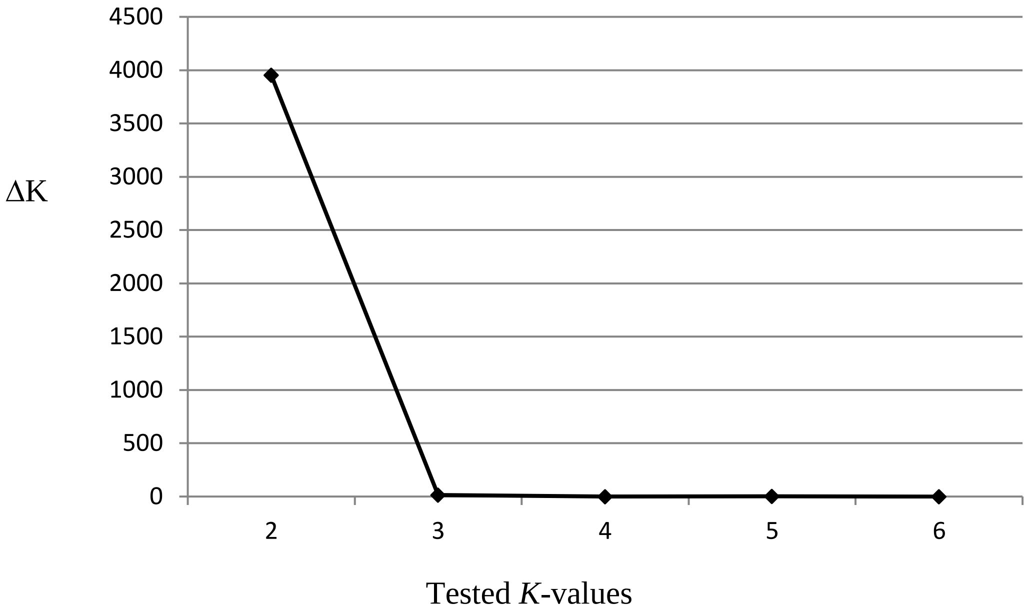

Figure S1. Δ*K* plotted as a function of the number of clusters with *K* ranging from 1 to 7.

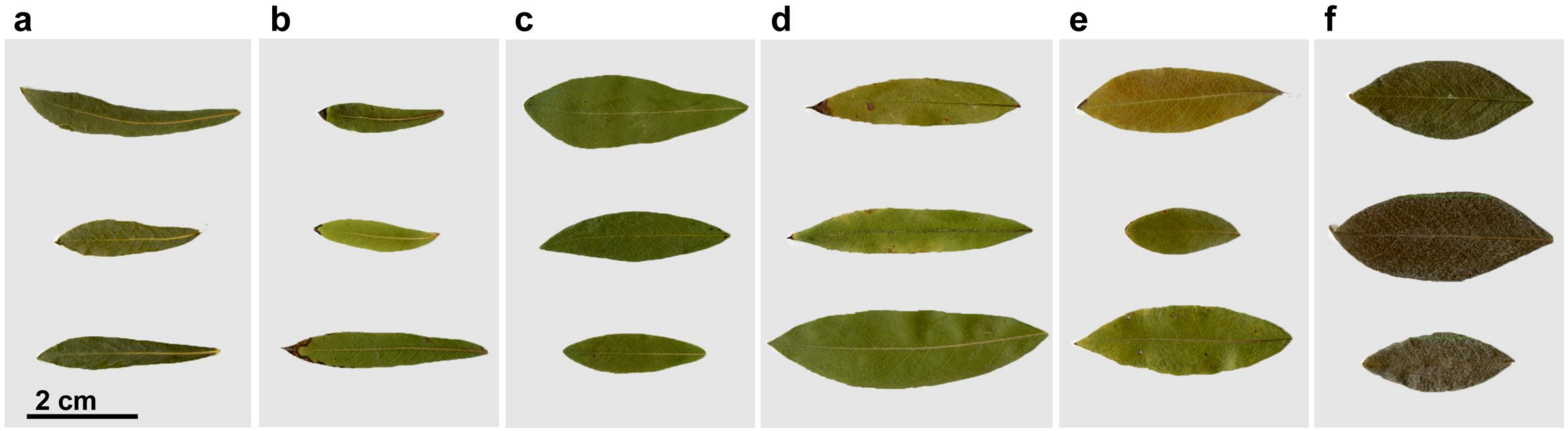

Figure S2. Examples of the leaf shape of *S. purpurea* (a), *S. helvetica* (f), F_1 hybrids (c), F_2 hybrids (d), and some backcrosses to *S. purpurea* (b) and backcrosses to *S. helvetica* (e) that showed marked resemblance to the recurrent parent. The scale is the same in all pictures.

Table S1. Admixture proportions (q_i) of the 40 remaining F_1 hybrids based on two clusters (K=2) estimated by STRUCTURE.

F1 individual	*S. purpurea* cluster	*S. helvetica* cluster
Hybrid1	0.541	0.459
Hybrid2	0.548	0.452
Hybrid3	0.544	0.456
Hybrid4	0.546	0.454
Hybrid5	0.536	0.464
Hybrid7	0.532	0.468
Hybrid9	0.543	0.457
Hybrid10	0.536	0.464
Hybrid11	0.540	0.460
Hybrid12	0.538	0.462
Hybrid13	0.536	0.464
Hybrid14	0.537	0.463
Hybrid15	0.540	0.460
Hybrid17	0.545	0.455
Hybrid18	0.536	0.464
Hybrid19	0.540	0.460
Hybrid20	0.552	0.448
Hybrid21	0.546	0.454
Hybrid22	0.542	0.458
Hybrid23	0.539	0.461
Hybrid24	0.540	0.460
Hybrid25	0.549	0.451
Hybrid26	0.538	0.462
Hybrid28	0.544	0.456
Hybrid29	0.538	0.462
Hybrid30	0.540	0.460
Hybrid31	0.552	0.448
Hybrid32	0.542	0.458
Hybrid33	0.535	0.465
Hybrid34	0.548	0.452
Hybrid35	0.543	0.457
Hybrid36	0.552	0.448
Hybrid37	0.535	0.465
Hybrid39	0.540	0.460
Hybrid40	0.543	0.457
Hybrid41	0.543	0.457
Hybrid42	0.539	0.461
Hybrid43	0.550	0.450
Hybrid44	0.540	0.460
Hybrid45	0.536	0.464

Table S2. Assignment of the hybrid class of the remaining 40 F_1 hybrids in NewHybrids. The table shows the estimated posterior probabilities that an individual belongs to each of the six different genotype frequency classes.

F1 individual	*S. purpurea*	*S. helvetica*	F1	F2	Backcross to *S. purpurea*	Backcross to *S. helvetica*
Hybrid1	0	0	1	0	0	0
Hybrid2	0	0	1	0	0	0
Hybrid3	0	0	1	0	0	0
Hybrid4	0	0	1	0	0	0
Hybrid5	0	0	1	0	0	0
Hybrid7	0	0	1	0	0	0
Hybrid9	0	0	1	0	0	0
Hybrid10	0	0	1	0	0	0
Hybrid11	0	0	1	0	0	0
Hybrid12	0	0	1	0	0	0
Hybrid13	0	0	1	0	0	0
Hybrid14	0	0	1	0	0	0
Hybrid15	0	0	1	0	0	0
Hybrid17	0	0	1	0	0	0
Hybrid18	0	0	1	0	0	0
Hybrid19	0	0	1	0	0	0
Hybrid20	0	0	1	0	0	0
Hybrid21	0	0	1	0	0	0
Hybrid22	0	0	1	0	0	0
Hybrid23	0	0	1	0	0	0
Hybrid24	0	0	1	0	0	0
Hybrid25	0	0	1	0	0	0
Hybrid26	0	0	1	0	0	0
Hybrid28	0	0	1	0	0	0
Hybrid29	0	0	1	0	0	0
Hybrid30	0	0	1	0	0	0
Hybrid31	0	0	1	0	0	0
Hybrid32	0	0	1	0	0	0
Hybrid33	0	0	1	0	0	0
Hybrid34	0	0	1	0	0	0
Hybrid35	0	0	1	0	0	0
Hybrid36	0	0	1	0	0	0
Hybrid37	0	0	1	0	0	0
Hybrid39	0	0	1	0	0	0
Hybrid40	0	0	1	0	0	0
Hybrid41	0	0	1	0	0	0
Hybrid42	0	0	1	0	0	0
Hybrid43	0	0	1	0	0	0
Hybrid44	0	0	1	0	0	0
Hybrid45	0	0	1	0	0	0
